# PRAME positive spiradenocarcinoma treated with Mohs micrographic surgery

**DOI:** 10.1016/j.jdcr.2025.01.022

**Published:** 2025-02-17

**Authors:** Austinn C. Miller, John S. Acosta-Peñaloza, Monica Constantinescu, András Schaffer, Armand B. Cognetta

**Affiliations:** a Dermatology Associates of Tallahassee, Tallahassee, Florida; b University of Central Florida/HCA Healthcare Consortium, Tallahassee, Florida; c Florida State University College of Medicine, Tallahassee, Florida; d Division of Dermatology, Mohs Micrographic Surgery Unit, Florida State University College of Medicine, Tallahassee, Florida

**Keywords:** malignant eccrine spiradenoma, Mohs Micrographic Surgery, PRAME, spiradenocarcinoma

## Introduction

Spiradenocarcinoma, or malignant eccrine adenoma, is an exceedingly rare adnexal tumor. Typically affecting elderly individuals, this tumor commonly presents as a nondistinct painless mass. Diagnosis relies upon histopathology, which may pose considerable challenges given its varied patterns.^1^ Accurate distinction from other adnexal tumors, especially those with squamous differentiation, necessitates a meticulous histopathologic assessment supplemented by immunohistochemical analyses. Preferentially Expressed Antigen in Melanoma (PRAME) immunohistochemical findings have not been described previously in spiradenocarcinoma. Herein, we present a case of spiradenocarcinoma, with a comprehensive histologic analysis demonstrating PRAME positivity, as well as treatment with Mohs Micrographic Surgery (MMS).

## Case presentation

**Fig 1:**
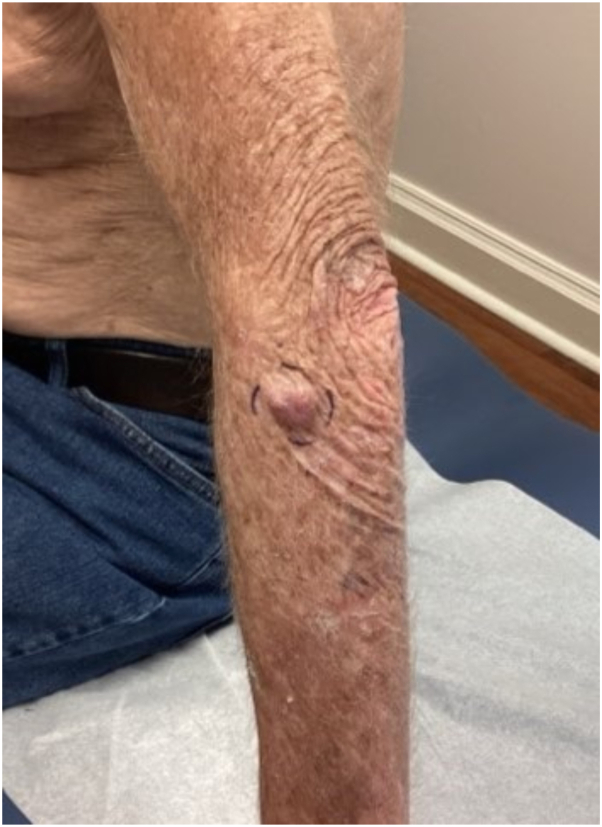
Firm, mobile, irregular, 2 cm dermal/subdermal nodule with a dusky red hue on the radial aspect of the left forearm.

An 82-year-old male with an extensive history of nonmelanoma skin cancer (squamous cell carcinoma and basal cell carcinoma) and melanoma in situ presented with a 2 × 2 cm firm, mobile, subcutaneous nodule with a dusky red hue on the radial aspect of the left forearm (Fig 1). The clinical differential diagnosis of this solitary subcutaneous nodule was broad; however, a malignant process was favored given its irregular appearance, firmness, and the patient’s nonmelanoma skin cancer history.

**Fig 2:**
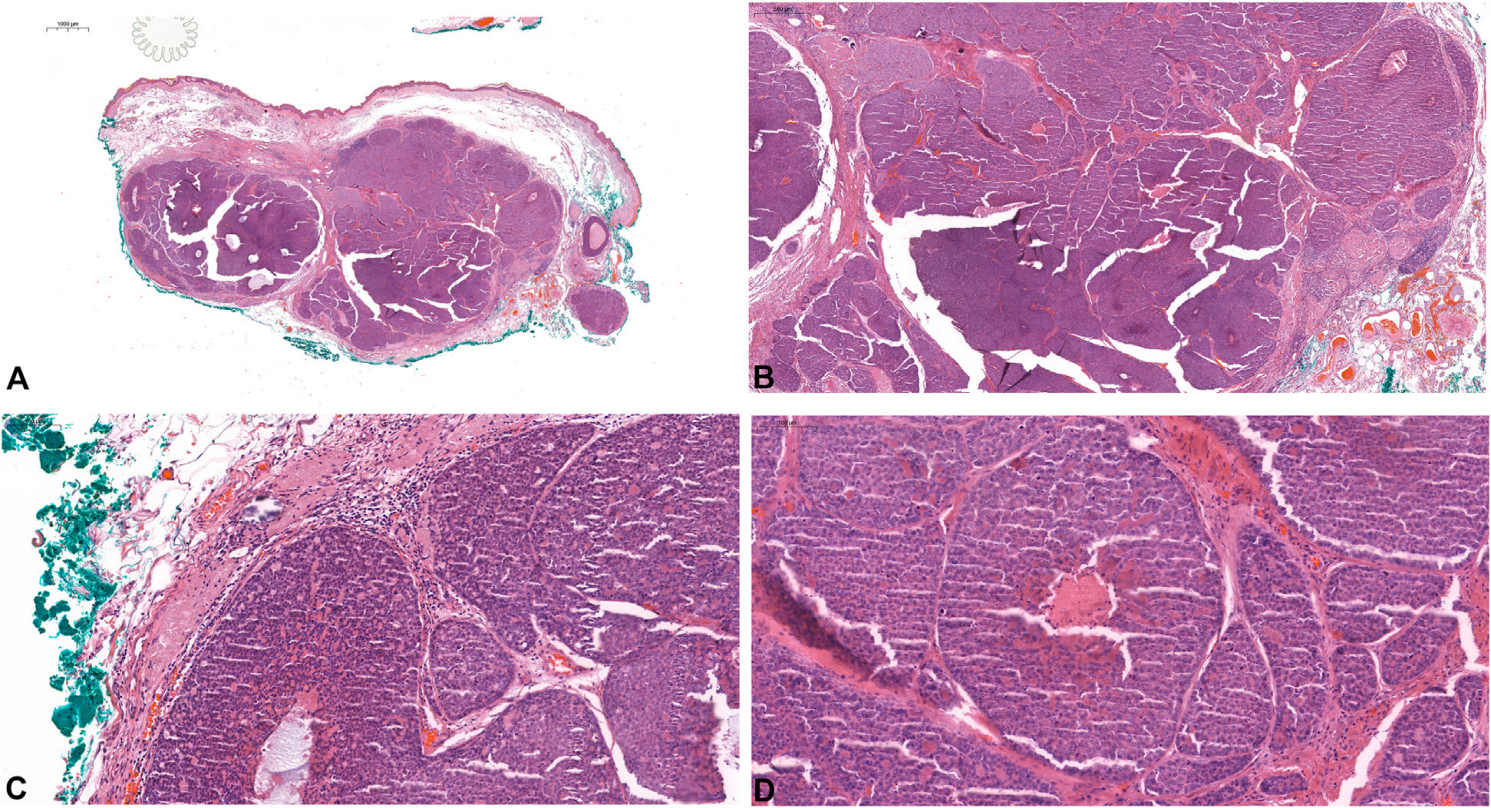
**A,** Excisional biopsy shows well-circumscribed multinodular structure in pushing downward on subcutis. H&E, 2×. **B,** Higher power view demonstrates fascicles separating dense cellular islands. H&E, 10×. **C** and **D,** Architecture recapitulates that of a benign spiradenoma, with 2 types of basaloid cells: small, dark blue nuclei and large, pale blue nuclei, some forming circular rosettes of basaloid cells arranged around pink hyalinized stroma and intermixed with small sweat ducts. However, tumor islands show increased mitotic figures, nuclear pleomorphism, and prominent nucleoli. Focal comedo necrosis is visible, highlighting malignant change. H&E, 20×.

**Fig 3:**
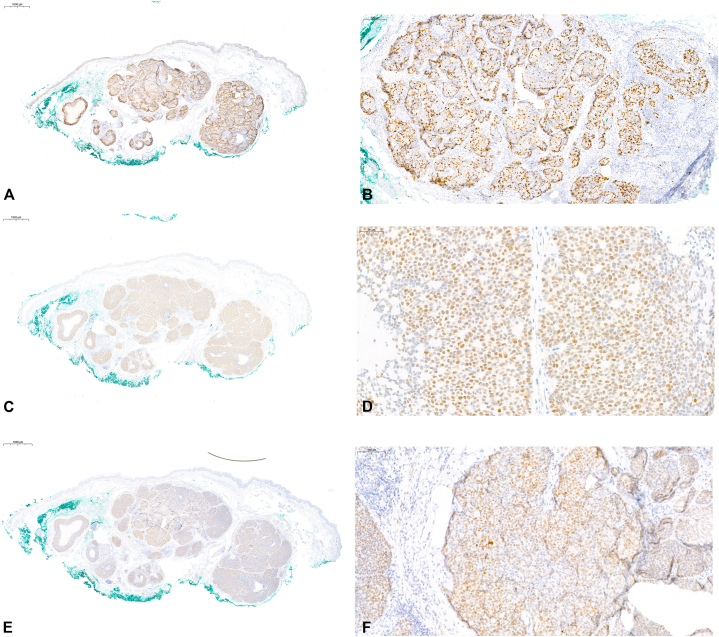
**A,** Increased Ki-67 proliferation index, up to 40% in malignant areas. **B,** Ki-67 positivity in atypical lesional cells. **C,** p53 expression is positive in carcinomatous areas (nuclear stain). **D,** p53 is over-expressed within malignant cells. **E,** PRAME shows >70% positivity in lesional cells. **F,** PRAME expression in malignant areas.

Excisional biopsy revealed well-circumscribed nodules deep in the dermis/subcutis, devoid of epidermal connection (Fig 2). Microscopically, the nodules exhibited a highly cellular tumor composition, characterized by 2 distinct types of basaloid cells: cells with small, dark blue nuclei admixed with large, pale blue nuclei containing cells. Additionally, small sweat ducts and circular rosettes of basaloid cells arranged around hyalinized stroma were observed. Tumor islands displayed increased mitotic activity, nuclear pleomorphism, prominent nucleoli, and focal comedo necrosis. Immunohistochemical analysis revealed an elevated Ki-67 proliferation index, reaching up to 40% in malignant regions. Furthermore, p53 expression was discernible within carcinomatous areas. Notably, PRAME exhibited significant positivity, exceeding 70% in lesional cells (Fig 3). These histologic features supported a diagnosis of spiradenocarcinoma over atypical spiradenoma or cylindrocarcinoma.

Following the histologic evaluation, the patient underwent slow MMS with permanent sections, achieving clearance after one stage.

## Discussion

The estimated incidence of spiradenocarcinoma is 0.07 cases per million person-years.^2^ As such, malignant transformation of eccrine spiradenoma is an extremely rare occurrence and de novo formation is even less frequent.^2^^,^^3^ The exact factors that trigger this transformation are not fully understood, but it is believed that genetic alterations and environmental factors may play a role.^4^ Our patient may have been predisposed to developing spiradenocarcinoma given his Fitzpatrick II skin type and extensive evidence of UV-damage including a history of numerous NMSCs and melanoma in situ. While UV-damage has not been directly linked with spiradenocarcinoma, it likely plays a role, as they are more commonly encountered in lighter skin types.^2^

The mean age at diagnosis of spiradenocarcinoma is 62.8 years, with cases ranging from 25 to 99 years and a median age of 63 years.^2^ Gender distribution is relatively equal, with 47.8% of cases occurring in males and 52.2% in females. Among ethnic groups, spiradenocarcinoma cases are predominantly observed in White patients, accounting for 74% of the cohort. The distribution among other ethnicities includes 11.1% black, 6.7% Hispanic, and 4.4% Asian/Pacific Islander.^2^

Most cases of spiradenocarcinoma are reported on the head, neck, and trunk.^2^ Location of the tumor on the extremities is less commonly reported as in our case.

Histologically, spiradenocarcinoma is characterized by increased cellularity, nuclear pleomorphism, increased mitotic activity, and the presence of necrosis, all of which are indicative of malignant transformation.^2^ In unclear cases, immunohistochemistry can be used to assist diagnosis. Typical stains include Ki-67, p53, MYB, CK5, CK6, CK7, CEA, and EMA.^5^ PRAME is a cancer-testis antigen involved in the regulation of tumor-specific immune responses. It is expressed in a variety of malignancies but not normal tissues. Therefore, it is often used for cancer detection and prognostic purposes. The use of PRAME has not been described in spiradenocarcinoma, although its use in sebaceous adnexal tumors has been demonstrated. Furthermore, adnexal tumors of sweat gland origin typically stain negative for PRAME, including spiradenoma.^6^ Thus, PRAME may serve as a potential diagnostic marker in spiradenocarcinoma.

Most reported cases have been treated with wide local excision with >1 cm margins without adjuvant radiation.^2^ Few cases have been treated with MMS.^2^ In this case, we chose to perform slow MMS with permanent sections, given the unusual and aggressive nature of this tumor. We recommend MMS as the preferred treatment option when feasible, given its superior effectiveness, lower recurrence rates, tissue-sparing advantages, and potential for improved disease-specific survival compared to other treatment modalities in malignant adnexal tumors.^7^^,^^8^

While most spiradenocarcinomas remain localized (66%), local recurrence and metastasis have been reported, making it a potentially life-threatening condition.^2^ Metastasis most often develops in regional lymph nodes with involvement noted in 2.2% of cases upon initial diagnosis, while distant metastatic disease is identified in 3.3% of cases at the time of diagnosis.^2^^,^^5^ Sites of distant metastasis include bones, lungs, brain, and liver.^5^ In cases of metastatic disease, the role of adjuvant therapies such as chemotherapy, radiotherapy, and immunotherapy remains less defined. However, there are reports of using PD-1 inhibitors like pembrolizumab in metastatic settings.^9^ Close follow-up is essential to detect possible recurrence or metastatic disease. Patients should be followed every 3 months for the first year after diagnosis then every 6 months in the second year then annually thereafter.^10^ No consensus exists for laboratory studies or routine imaging, but annual chest radiographs, liver function tests, liver ultrasound, chest and abdomen CT, and/or MRI may be useful.

## Conflicts of interest

None disclosed.
